# The compassionate love for humanity scale (CLS-H-SF): psychometric properties of the Persian version

**DOI:** 10.1186/s40359-022-00776-x

**Published:** 2022-03-12

**Authors:** Zahra Hajiheydari, Abbas Abdollahi, Saade Abdalkareem Jasim, Tawfeeq Abdulameer Hashim Alghazali, Supat Chupradit, Caomhán McGlinchey, Kelly A. Allen

**Affiliations:** 1 Department of Counseling, Faculty of Education and Psychology, Alzahra University, Tehran, Iran; 2 Department of Medical Laboratory Techniques, Al-Maarif University College, Ramadi, Iraq; 3 English Department, The Islamic University, Najaf, Iraq; 4 Department of Occupational Therapy, Faculty of Associated Medical Sciences, Chiang Mai University, Chiang Mai, 50200 Thailand; 5 Graduate School of Education, University of Exeter, Exeter, UK; 6 School of Educational Psychology and Counselling, Monash University, Melbourne, Australia

**Keywords:** Compassionate love, Compassion, Persian, Instrument, Validity, Reliability

## Abstract

Compassionate love is beneficial in a variety of domains, including in education, health, and law, as well as in people’s personal lives. The topic of compassionate love has therefore attracted growing interest from researchers interested in its psychological and social dimensions. Given the importance of compassion to the education and health sectors, and the expansion of these sectors in Iran, this paper aims to provide Persian (Farsi) speaking practitioners and researchers with an effective instrument for measuring compassion. As such, the authors have translated the compassionate love for humanity scale-short form (CLS-H-SF) into the Persian language, and assessed the psychometric properties of this instrument among a sample of the Iranian population. A sample of 827 adults (49.9% women and 51.1% men) completed the Persian version of the CLS-H-SF through an online survey. Concurrent validity was assessed using the Persian versions of the positive and negative affect scale, self-esteem scale, and satisfaction with life scale. The CLS-H-SF positively correlated with positive affect, self-esteem and life satisfaction, and negatively correlated with negative affect. These findings indicate acceptable concurrent validity for the CLS-H-SF. Cronbach’s alpha for the scale was 0.88, indicating good internal consistency between items. A confirmatory factor analysis supported a one-factor model same as the English version of the CLS-H-SF. The findings of this study showed the Persian version of CLS-H-SF had acceptable validity and reliability in assessing compassionate love for humanity in Iranian adults.

## Introduction

For over three decades, researchers have grown increasingly interested in the subject of compassion [1–4]. The benefits of compassion have been highlighted by world-renowned institutions in the fields of healthcare, education and law [5]. Studies have shown individuals with high levels of compassion are more likely to show pro-social behaviors and altruistic behaviors [5, 6]. Also, studies have shown that people with higher levels of compassion experience less perfectionism, stress and depression [7, 8]. However, compassion is not merely appreciated for its practical benefits. Instead, it is celebrated by many of the world’s most prominent religious traditions [5]. Demonstrating compassion is therefore considered by many to be a morally necessary human endeavor [9, 10]. Therefore, measuring compassion can help to assess the amount of compassion in individuals and help professionals to have a more accurate identification in employing people choosing job placement, which need high levels of compassion, as well as performing interventions to improve compassion in individuals.

Compassion has long held an elevated position in human culture, and so debates surrounding the meaning and value of compassion can be dated back more than 2000 years [11]. This is important to note because it means that translating a tool for measuring compassion is not simply a matter of translating from one language to another. Instead, different cultures are likely to have arrived at nuanced views of compassion; views which will most likely have implications for the way in which a tool for measuring compassion is used and understood. For example, much contemporary research on compassion begins with the Buddhist view of the construct. In this tradition, compassion is understood to be “*compassionate love*” or “*loving-kindness*”, and its purpose is to alleviate the suffering of others [12–14]. Consistent with Buddhism concepts, self-compassion means the compassion for others [15]; however, it should note that the compassionate love is the more comprehensive construct. As such, although compassionate love associates with empathy, compassionate love distinguishes from empathy. Compassionate love is a tendency towards others (i.e., acquaintances, or strangers, or humanity as a whole), especially when they are suffering or in need [16]. As defined by McDonald et al. [15], compassionate love is a long-term characteristic that includes empathetic feelings as well as patterns of action toward other people. However, Farsi-speaking practitioners and researchers are more likely to have encountered compassion, as it is understood in the Islamic tradition. As such, while the loving-kindness aspect is represented by the *ihsan* (benevolence) aspect of Islamic compassion, there are two additional aspects—*rahma* (mercy) and *adl* (fairness/justice)—which may also occur to Farsi speakers [17]. Of course, a diverse understanding of compassion across cultures is not in and of itself a problem. However, a universally agreed upon understanding of the construct may be useful for researchers and practitioners who wish to communicate their work to one another meaningfully and effectively; especially if they come from a diverse range of cultural and disciplinary backgrounds. An agreed upon definition of compassion may be a useful first step.

Compassion for others, then, has been defined as “*a feeling of concern and a desire to help when exposed to others’ suffering*” [15], however, the construct has been conceptualized in a variety of ways [5]. Researchers adhering to the Buddhist view of compassion have opted to measure a slightly broader construct than that defined by McDonald et al. [15]. Other researchers have focused on the altruistic aspect of compassion, conceptualizing compassion as a more enduring, trait-like variable than might be suggested by McDonald et al.’s definition. Both Hwang et al. [18] and Sprecher and Fehr [16] have focused on compassion as a pattern of behavior, such as self-sacrificing tendencies, which emerge in an individual due to the empathic feelings they have toward different groups of people.

Researchers interested in empathy, meanwhile, have focused on the cognitive and affective components of concern for others’ suffering [19] i.e., they have explored which empathetic thoughts and feelings coincide with compassion, but have been less interested in the compassionate behavior which may follow these thoughts and feelings. In fact, some researchers have been keen to distinguish between compassion and empathy, especially given that these constructs can lead to different mental health outcomes [5]. This is because simply sharing negative emotions has been shown to have negative psychological consequences when individuals are not offered support to process these emotions [15]. Nevertheless, empathy has been shown to be an important component of compassion as a construct [18].

In terms of trying to operationalize compassion, an important contribution was made when Sprecher and Fehr [16] published their 21-item Compassionate Love for Humanity Scale (CLS-H) in 2005. It was, in fact, one of the first instruments to measure compassion, and it focused largely on the altruistic component of the construct. Not long afterwards, Hwang et al. [18] developed a 5-item scale called the Santa Clara Brief Compassion Scale (SCBCS), based on the original CLS-H. This short scale was designed with large, multivariate studies in mind, and was designed to be quick to administer because these studies include large groups of participants who will have a variety of additional tests and scales administered to them [20]. A decade later, Chiesi et al. [21] revised the CLS-H itself, developing the 9-item compassionate love for humanity scale-short form (CLS-H-SF). For Chiesi et al. [21], this tool improved on the SCBCS in a number of ways, including by preserving “items that provided the most information” in order to “maximize [the tool’s] effectiveness” (p. 7).

In summary, research on compassion is both necessary and growing. Its importance to the healthcare, education and legal systems has been highlighted by a number of world-renowned institutions (Strauss et al. [5]). An effective tool is therefore necessary to measure this construct [21]. This is especially the case in Iran, where the healthcare, education and legal systems are developing at a rapid pace [22, 23]. As such, a tool for measuring compassion that can be used by researchers and practitioners in their native Persian language is warranted. The present study therefore aimed to measure the reliability and validity of the Persian version of the CLS-H-SF scale. This study also used measures that were employed to assess convergent and divergent validity in the original psychometric study of Chiesi et al. [21]. This study hypothesized that the scores of the positive affect schedule (PAS; [24], the self-esteem scale (SES) [25] and satisfaction with life scale (SWLS) [26] would positively correlate with the Compassionate Love for humanity scale-short form (CLS-H-SF). We also hypothesized that the negative affect schedule (NAS) [24] would negatively correlate with the compassionate love for humanity scale-short form (CLS-H-SF).

## Method

### Participants

Participants were 827 Iranian adult volunteers (413 women; 49.9%) with an age range of between 18 and 65 years (*M* = 32.9, *SD* = 8.4). In terms of education level, 44 (5.3%) participants had less than a diploma, 168 (20.3%) had a diploma, 376 (45.5%) had a bachelor’s degree, 186 (22.5%) held a master’s degree, and 53 (6.4%) had a doctorate degree. A total of 839 volunteers completed questionnaires, however the data from 12 participants was removed, because Mahalanobis D2 values were greater than 4 [27]. Due to the use of an online survey and setting the necessary answers for each item, there was no missing data. A total of 827 responses were used in the data analysis.

### Procedure

All participants were unpaid volunteers, therefore obtaining informed consent was a priority. Consenting participants and questionnaires were then entered into *Google Forms* and the link was sent to social networks to be completed online by the respondents. The survey collected demographic characteristics, including age, education, and gender. The survey also included an information sheet reminding participants that their participation was both voluntary and anonymous. The data collection period began in May 2021 and ended in October 2021. The online questionnaires took an average of 40 min to complete for each respondent. Also, the procedure of the study was approved by an ethical committee of Alzahra University (IR/10/27/1400). All procedures were carried out in accordance with applicable guidelines and regulations. Alongside the translated CLS-H-SF, the additional scales used in this study were already translated into Persian.

To translate the English version of CLS-H-SF to Persian, Brislin [28] back-translation method was used. Two English-Persian translators whose first language was Persian conducted the translation. The first translator is tasked with translating the *original source language document* from the source language (in this case English) into the target language (Persian), creating a *draft target language document*. The second translator then blindly translates the *draft target language document* from the target language (Persian), back into the source language (English), creating a *copy source language document*. The two source language documents can then be assessed for discrepancies and inconsistencies. This process can then be iterated until there are as few discrepancies between the source language documents as possible [29]. The third translator, who was fluent in Persian and English language, compared the two translated versions and finalized the Persian version.

### Preliminary data analysis: face validity

Before the data was analyzed, preliminary tests of validity were carried out on the Persian translation of the instrument. First the instrument was assessed for face validity, which is the degree to which end users agree that the items of an assessment instrument appropriately reflect the targeted construct—in this case compassion. Face validity was assessed in two ways. First, it was assessed qualitatively by following Boateng et al. [30] recommendations. Telephone interviews were therefore carried out with ten Persian-speaking lay colleagues, each of whom was asked whether they agreed that the items represented a facet of compassionate love.

Face validity was next assessed quantitatively, as this can be a useful way of determining whether an item is excessively difficult, ambiguous or relevant [31]. Face validity was assessed quantitatively by surveying 10 participants to determine the “*impact score*” of each item. The impact score for each item is calculated by multiplying the “*importance*” of the item, as determined by respondents, by the frequency of these responses [32]. In this context, “*importance*” means the level to which respondents agree an item reflects the construct, and “*frequency*” is the number of responses.

### Preliminary data analysis: content validity

Content validity was also assessed using both qualitative and quantitative phases. During the qualitative phase, ten colleagues experienced in the use of qualitative techniques, and familiar with the compassion literature assessed all nine items. In order to assess the content validity quantitatively, the item content validity index (I-CVI) and the item content validity ratio (I-CVR) were calculated [33]. Experts evaluate the items by selecting one of the values from (1) “*not relevant at all”* to (4) “*highly relevant”*. The value of the I-CVI is calculated by dividing the number of experts who selected ‘3’ and ‘4’ by the total number of experts. The I-CVR estimates the essentiality of items from the experts' point of view. Experts evaluate the items by selecting a value from (1) “*not essential”* to (3) “*essential”*. The value of I-CVR is calculated as follows: the number of experts who select the value of ‘3’ minus half the number of experts is divided by half the number of experts. According to Polit et al. [34], a I-CVI greater than 0.7 indicates acceptable content validity. For ten experts, a I-CVR value greater than 0.62 indicates acceptable content validity [35]. Similarly, the scale content validity index (S-CVI) is calculated by counting the number of items in a measure that have received a *"highly relevant*" rating. S-CVI can be calculated using two methods: the Universal Agreement (UA) among experts (S-CVI/UA) and the Average CVI (S-CVI/Ave), the latter being a less cautious method. S-CVI/UA is computed by adding all items with I-CVI equal to 1 and dividing by the total number of items, whereas S-CVI/Ave is calculated by summing the I-CVIs and dividing by the total number of things. Excellent content validity is demonstrated by S-CVI/Ave equal or greater than 0.9 [36].

### Data analysis proper: construct validity

*SPSS* software and *AMOS-24* software were used to analyze data and calculate descriptive statistics. In order to determine whether the Persian CLS-H-SF shared the same single-factor structure as Chiesi et al.’s [21] English language counterpart, the factor structure of the scale was explored using a confirmatory y factor analysis (CFA). According to Kline [37], factor loadings should be discarded if they are negative values; or if they are less than 0.40, or greater than 1.0. average variance extracted (AVE) was also used to evaluate the convergent validity of the scale. AVE values above 0.4 are considered acceptable [38]. Internal consistency was explored using both a construct reliability (CR) test and a Cronbach’s alpha test for reliability. CR values above 0.7 are felt to be acceptable [39], while a Cronbach’s alpha value above 0.7 is considered a good indicator of internal reliability in the social sciences [40].

### Data analysis proper: concurrent validity

In order to determine the concurrent validity of the CLS-H-SF, Chiesi et al. [21] calculated bivariate correlations for both the CLS-H and CLS-H-SF; and three additional instruments which measure related psychological constructs. These three instruments were the Positive and Negative Affect Schedule [24], the Self-Esteem Scale [25], and the Satisfaction with Life Scale [26]. As such, the authors of this study examined the concurrent validity of the Persian CLS-H-SF by carrying out similar statistical analyses between the Persian CLS-H-SF and the same three instruments.

## Measures

### Compassionate love for humanity scale-short form (CLS-H-SF)

The translated 9-item CLS-H-SF [21] was used to measure the participants’ compassionate love for humanity. The English version of the scale has demonstrated adequate validity and reliability, and good fit indices in a one-factor model [21]. Participants responded to each item on a 7-point Likert scale (*1* = *I strongly disagree to 7* = *I strongly agree)*. Each item on the scale is presented as a statement, for example, item 5 reads: “*I tend to feel compassion for people even though I do not know them*.”

### Positive and negative affect schedule (PANAS)

The PANAS [24] is a 20-item measure that assesses positive affect (PA) and negative affect (NA) on a 5-point Likert scale (*1* = *not at all to 5* = *extremely*). The scale requires participants to “indicate the extent you have felt this way over the past week”, before providing twenty different emotional states, including “excited” (item 3), “upset” (item 4) and “guilty” (item 6). Watson et al. observed Cronbach's alpha values of 0.88 for the positive affect aspect of the scale, and 0.87 for the negative affect aspect. Among an Iranian sample, meanwhile, Crocker [41] recorded Cronbach's alpha values of 0.85 for both for the positive and negative affect aspects of the scale.

### Self-esteem scale (SES)

The SES [25] is a 10-item measure that assesses self-esteem and personal worth on a 4-point Likert scale (*1* = *strongly disagree to 4* = *strongly agree*). The scale supports a two-factor structure and has an acceptable reliability with a Cronbach's alpha of 0.87 [42]. Each item on the scale is presented as a statement, for example, item 4 reads: “I am able to do things as well as most other people”.

### Satisfaction with life scale (SWLS)

The SWLS ([26]is a 5-item measure that assesses life satisfaction on a 7-point Likert scale (*1* = *Strongly disagree to 7* = *Strongly agree*). Reliability was obtained using the test–retest method (0.82) and Cronbach's alpha (0.87). Each item on the scale is presented as a statement, for example, item 5 reads: “The conditions of my life are excellent.” The Persian version has adequate psychometric properties, with a Cronbach's alpha of 0.83 [43].

## Results

### Face validity

All nine items on the scale were felt to reflect compassion by the ten respondents during the qualitative phase. During the quantitative phase, all nine items achieved an impact score greater than the lower threshold of 1.5 [32], and so all items were retained (Table 1).

**Table 1:** Impact scores for the items of CLS-H-SF scale

Item	Difficulty	Ambiguity	Relevancy
1. When I hear about someone (a stranger) going through a difficult time, I feel a great deal of compassion for him or her	4.14	4.05	4.05
2. It is easy for me to feel the pain (and joy) experienced by others, even though I do not know them	4.8	4.23	3.52
3. If I encounter a stranger who needs help, I would do almost anything I could to help him or her	4.05	4.7	3.96
4. I feel considerable compassionate love for people from everywhere	4.05	4.8	4.6
5. I tend to feel compassion for people even though I do not know them	4.7	4.8	4.7
6. One of the activities that provide me with the most meaning in my life is helping others in the world who need help	3.96	4.7	4.8
7. I often have tender feelings toward people (strangers) when they seem to be in need	4.8	4.7	4.23
8. I feel selfless caring for most of mankind	1.75	2.87	3.87
9. If a person (a stranger) is troubled, I usually feel extreme tenderness and caring	4.05	4.8	3.28

### Content validity

All items achieved a CVI greater than 0.7, indicating acceptable content validity (Table 2; Polit et al. [34]). A CVR value greater than was also observed, indicating acceptable content validity (see Table 2). Content validity was therefore acceptable for all nine items. Also, the value of S-CVI/Ave (0.937) was greater than 0.9.

**Table 2:** CVI and CVR values for the items of CLS-H-SF scale

Item	CVI			CVR
	Difficulty	Ambiguity	Relevancy	Urgency
Item1	1	0.9	0.8	0.8
Item2	0.9	0.9	1	1
Item3	1	0.9	1	1
Item4	1	0.9	0.9	0.8
Item5	1	0.9	1	1
Item6	1	0.9	1	1
Item7	1	0.9	1	0.8
Item8	0.7	0.9	0.9	0.6
Item9	1	0.9	1	0.8

### Construct validity

**Fig. 1:**
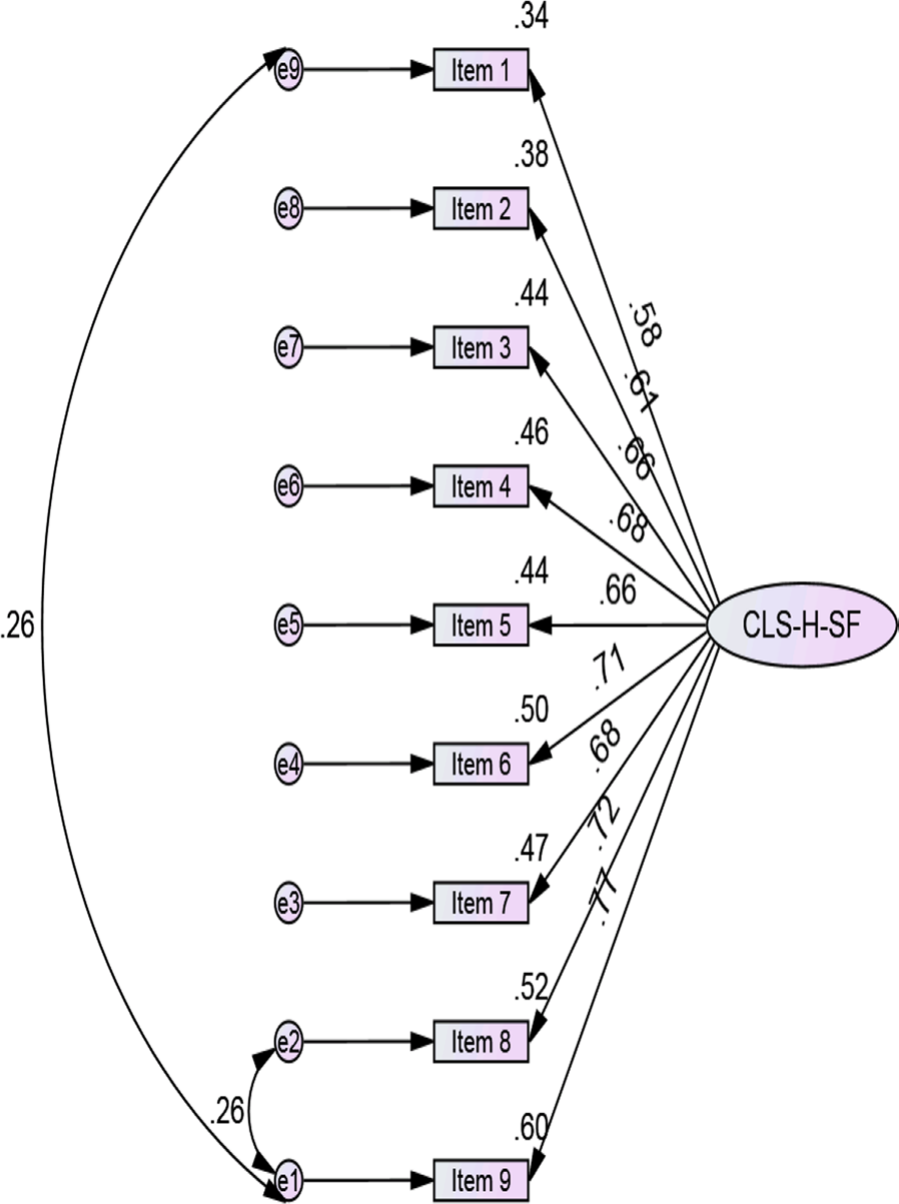
Confirmatory factor analysis of the CLS-H-SF scale

Factor loadings were greater than 0.5 and met the criteria outlined by Kline (2015; see Fig. 1). The CFA for the Persian CLS-H-SF among the Iranian participants therefore mirrored the one-factor structure of the English language CLS-H-SF [21], suggesting acceptable construct validity.

The acceptable cut-off scores for measurement fit indices are CMIN/DF (Chi-Square/Degree of Freedom) < 5; CFI (Comparative Fit Index) > 0.90; RMSEA (Root Mean Square Error of Approximation) < 0.08; TLI (Tucker-Lewis index) > 0.90; and GFI (Goodness of Fit Index) > 0.90 (Byrne, 2013). The findings revealed the model had appropriate measurement fit indices (CMIN/DF = 4.3, CFI = 0.97, RMSEA = 0.06, TLI = 0.96, GFI = 0.97).

The AVE indicated acceptable convergent validity (AVE = 0.46), while the CR value (0.86) exceeded the 0.7 cut-off suggested by Tabachnick et al. [44], indicating acceptable internal consistency. Finally, a Cronbach’s alpha value of 0.88 was reported for this scale, also indicating good internal consistency [40].

### Concurrent validity

Correlation analysis showed that the Persian CLS-H-SF was positively correlated with positive affect, self-esteem, and life satisfaction, and negatively correlated with negative affect (see Table 3).

**Table 3:** Correlations between the studied variables

Variables	1	2	3	4	5
(1) CLS-H-SF	1				
(2) PA	0.18*	1			
(3) NA	− 0.13*	− 0.11*	1		
(4) SES	0.15*	0.19*	− 0.12*	1	
(5) SWLS	0.17*	0.16*	− 0.11*	0.11*	1

*p* < 0.05*CLS-H-SF compassionate love for humanity scale-short form, *PA* positive affect, *NA* negative affect, *SES* self-esteem scale, *SWLS* satisfaction with life scale

## Discussion

Compassionate love represents a positive orientation towards others. It involves noticing that others are suffering, and then experiencing thoughts and feelings associated with this noticing which motivate helpful action [15]. Those who experience higher levels of compassionate love are expected to be more empathetic [18], because feelings of empathy are often those which motivate compassionate actions. However, unlike empathy, compassion is associated with positive action, rather than solely with positive feelings towards others [45]. Compassionate love, therefore, motivates prosocial behaviors toward one's family, companions, peers, and community [46]. As such, compassionate love has a key role to play in the helping professions, where suffering is most likely to be encountered. This is likely why institutions tasked with supporting those who are vulnerable highlight the importance of compassion [5].

An English language tool for measuring compassionate love among Canadian undergraduates has proven valuable [21], which is why the authors of this study explored the psychometric properties of the Persian version of the CLS-H-SF among Iranian adults. A back-translation method was used to translate Chiesi et al.’s [21] CLS-H-SF, which ensured the CLS-H-SF was translated reliably into Persian [28]. Tests for face validity were then carried out which showed that the translated CLS-H-SF had acceptable face validity. Appropriate CVI and CVR values also indicated acceptable content validity for each item on the scale. Cronbach’s alpha (0.88) and CR (0.86) coefficients, meanwhile, also showed acceptable internal consistency. According to the results of face validity, content validity and construct validity, it can be concluded that the compassionate love is a culturally universal concept and there is no different in understanding the compassionate love between people who are living in western culture and Iranian population.

The results of the concurrent validity assessment were also consistent with Chiesi et al.’s [21] findings, as they indicated CLS-H-SF was positively correlated with positive affect (*r* = 0.10, *p* < 0.05), self-esteem (*r* = 0.14, *p* < 0.05), and life satisfaction (*r* = 0.16, *p* < 0.05); and negatively correlated with negative affect (*r* =  − 0.14, *p* < 0.05). The findings in this study indicated that the Persian CLS-H-SF was positively correlated with positive affect (*r* = 0.18, *p* < 0.05), self-esteem (*r* = 0.15, *p* < 0.05), and life satisfaction (*r* = 0.17, *p* < 0.05); and negatively correlated with negative affect (*r* = -0.13, *p* < 0.05). This study therefore indicates that the translated CLS-H-SF for adults is a psychometrically valid and reliable measurement tool.

### Implication of study

There are varieties of potential uses for this tool. For example, the Persian CLS-H-SF could be useful for employers in the helping professions, as it might help to ensure that they find employees who feel sympathy for their clients, and who are motivated to action this sympathy in response to the needs of their clients. Dierendonck and Patterson [47] however, have argued that compassionate love should have a greater role to play across all of business and industry. This is because compassionate leaders and managers are better equipped to understand the needs of their staff, and to respond to these needs. As such, they are likely to engender greater wellbeing among their workforce, thereby increasing efficiency, and achieving better outcomes for their businesses and line managers. Employers may find a measurement tool helpful for selecting more compassionate employees, or for measuring the effectiveness of training aimed at increasing employees’ compassion for one another. Psychologists and counsellors to screen for individuals who are particularly low on the compassionate love scale might also use the tool. These individuals may be offered follow-up interventions to help develop their compassionate and pro-social behaviors.

## Limitations and conclusion

In order to fully evaluate the potential for this tool, however, several limitations should be considered. A randomized sampling technique was not used for this study, and therefore the participants’ responses may not be an accurate reflection of the target population. Another limitation may have been brought about by the COVID-19 pandemic. Participants involved in this study are likely to have suffered extended bouts of social isolation before completing the survey, which may have influenced the levels of compassionate love they felt towards others. On the one hand, they may have felt more compassionate love, because the social isolation engendered an “absence makes the heart grow fonder” effect. Alternatively, participants may have felt less compassionate love towards others due to the absence of the “mere exposure” effect [48]. Replication of this study once the impact of COVID-19 has been resolved may therefore be worthwhile. Another limitation of the study could be that survey can introduce social desirability and response bias. Other studies could recruit interview and longitudinal methods to address the desired social response. Nevertheless, despite these limitations, compassionate love has had a vital role to play across human culture and throughout human history, and this paper is likely to be a valuable contribution to compassionate love -based research and practice in the Persian-speaking world.

## Data Availability

The datasets analysed during the current study available from the corresponding author on reasonable request.
